# A Possible Tool for Guiding Therapeutic Approaches to Urinary Infections with *Klebsiella pneumoniae*: Analyzing a Dataset from a Romanian Tertiary Hospital

**DOI:** 10.3390/antibiotics13121170

**Published:** 2024-12-03

**Authors:** Dragos Stefan Lazar, Maria Nica, Daniel Romeo Codreanu, Alma Gabriela Kosa, Lucian L. Visinescu, Corneliu Petru Popescu, Ion Cristian Efrem, Simin Aysel Florescu, George Sebastian Gherlan

**Affiliations:** 1 “Dr Victor Babes” Clinical Hospital of Infectious and Tropical Diseases, “Carol Davila” University of Medicine and Pharmacy, 030303 Bucharest, Romania; dragos.lazar@umfcd.ro (D.S.L.); daniel.chirita@spitalulbabes.ro (D.R.C.); alma.kosa@spitalulbabes.ro (A.G.K.); corneliu.popescu@umfcd.ro (C.P.P.); simin.florescu@umfcd.ro (S.A.F.); george.gherlan@umfcd.ro (G.S.G.); 2 Department of Information Systems & Analytics Austin, Texas State University, San Marcos, TX 78666, USA; llv19@txstate.edu; 3 Internal Medicine Department, Craiova University of Medicine and Pharmacy, 200349 Craiova, Romania; cristian.efrem@umfcv.ro

**Keywords:** carbapenem-resistant *Klebsiella pneumoniae*, urinary tract infection, risk estimation tool, carbapenemases, antibiotic therapy

## Abstract

**Introduction:** The emergence of carbapenem-resistant pathogenic bacteria is a growing global public health concern. Carbapenem-resistant uropathogenic strains of *Klebsiella pneumoniae* can cause uncomplicated or complicated urinary tract infections, leading to a high risk of treatment failure and the spread of resistance determinants. The objectives of this 24-month study were to identify the prognostic characteristics of patients who were infected with carbapenem-resistant *Klebsiella pneumoniae* (*CRKpn*) and to create a tool to estimate the probability of a *CRKpn* infection before having the complete results of a patient’s antibiogram. **Results:** We found that 41.6% of all urinary infections with *Kpn* were caused by *CRKpn*. Factors such as male gender, the presence of upper urinary tract infections, invasive urinary maneuvers, recent infection with or carriage of the germ, and the nosocomial occurrence of UTIs with *Kpn* were predictive for *CRKpn* infection. Based on these factors, we proposed a model to estimate the presence of *CRKpn*. **Methods:** A retrospective case–control study including all hospitalized patients with urinary tract infections (UTIs) caused by *Klebsiella pneumoniae* was carried out. We reported data as percentages, identified independent predictors of the presence of *CRKpn*, and proposed a tool to evaluate the probability through multivariate analysis. **Conclusions:** Through this study, we aim to provide clinicians with a tool to support decision making regarding first-line antibiotic treatment.

## 1. Introduction

The emergence and spread of carbapenem-resistant pathogenic bacteria is a growing global public health concern. Of these, *Klebsiella pneumoniae* (*Kpn*) is currently recognized as one of the most important Gram-negative bacteria responsible for healthcare-associated infections (HAIs). In fact, *Kpn* was one of the first bacteria that were discovered to cause infections in immunocompromised people. It is thought to be responsible for one-third of all infections caused by Gram-negative bacteria, such as urinary tract infections, pneumonia, liver abscesses, endophthalmitis, surgical wound infections, endocarditis, and sepsis [1,2,3]. Infections caused by this organism are associated with high mortality, prolonged hospitalization, and high costs [4]. The risk of *Kpn* causing a difficult-to-treat infection is exacerbated by the presence of virulence and resistance determinants on mobile genetic elements. As with other *Enterobacteriaceae*, this leads to a dramatic increase in the incidence of multidrug-resistant (MDR) or extensively drug-resistant (XDR) pathogens, as these bacteria are ubiquitous natural inhabitants of the human and animal microbiome [5]. Initially, *Kpn* infections were only described in humans, but in recent decades, cases in animals have also been reported. In addition to being a common pathogen in humans and mammals, *Kpn* is an important pathogen in several avian species [6,7,8]. The increasing number of reports from veterinarians of resistance to several classes of antibiotics, including carbapenems, and the occurrence of carbapenem-resistant *Klebsiella pneumoniae* (*CRKpn*) infections in the human community highlight the difficulty of controlling this scourge and the need to coordinate human and veterinary interventions to control these infections [9,10,11]. *Klebsiella pneumoniae* has numerous mechanisms by which it manages to evade the action of antibiotics. Some strains have become hypervirulent, particularly the K1 serotype of *Kpn*, which has been involved in the occurrence of liver abscesses and biliary tract infections [12]. Tolerance and persistence have long been recognized as helping bacteria survive antibiotic exposure [13]. Antibiotic resistance is produced in several ways in *Kpn*: the enzymatic inactivation and modification of antibiotics, the alteration of antibiotic targeting, mutations in porins, the increased expression of antibiotic efflux pumps, and biofilm formation [14]. *Klebsiella pneumoniae* possesses intrinsic resistance mechanisms, making it naturally resistant to penicillin G and ampicillin. Its ability to efficiently acquire resistance through plasmids and transposons has accelerated the accumulation of antibiotic resistance, particularly with the introduction of new antibiotic classes. The emergence of *Kpn* strains producing extended-spectrum beta-lactamases (ESBLs) highlights the plasmid-based resistance mechanisms that are encoded within the accessory genome. The development of broad-spectrum β-lactam antibiotics led to the rise of plasmid-mediated AmpC β-lactamases, persistent oxacillin resistance, and the proliferation of CTX-M β-lactamases, conferring resistance to second-, third-, and fourth-generation cephalosporins [15]. Consequently, ESBL-producing *Kpn* has become widespread globally, with its endemic prevalence reaching up to 50% in certain regions [16]. Following the introduction of carbapenems in the mid-1980s as “superantibiotics” targeting plasmid-mediated β-lactamases, resistance rapidly emerged with enzymes such as New Delhi metallo-β-lactamase-1 (NDM-1), carbapenemase KPC, and OXA-48. These enzymes drastically reduced the efficacy of these last-resort antibiotics [17]. The gene encoding KPC, located on the Tn440 transposon, was first identified in *Kpn* in 1996 in a patient in Brooklyn, New York, and subsequently spread globally [18]. Over the past decade, carbapenem-resistant *Klebsiella pneumoniae* (*CRKpn*) strains producing NDM have been reported in patients across the globe [19]. The oxacillinase OXA-48, initially isolated in Turkey in 2001, is now endemic there and has spread to Europe, Asia, the Americas, and the Middle East [20]. In *Kpn*, carbapenem resistance is primarily associated with β-lactamase production, including class A (KPC), class B metallo-β-lactamases (e.g., NDM-1, IMP, VIM), and class D oxacillinases (e.g., OXA-48). This resistance often coincides with porin loss, which is caused by mutations in porin-encoding or regulatory genes, and the overproduction of efflux pumps. Various carbapenemases have been identified in *Kpn*, including KPC (class A), NDM (class B), and OXA-48-like enzymes (class D), such as OXA-181 and OXA-244. Notably, strains producing KPC, OXA-48-like enzymes, or NDM-1 are often considered pan-resistant [21]. In contrast, IMP-1- and VIM-producing strains remain susceptible to specific agents such as ceftazidime–avibactam or meropenem–vaborbactam [22]. Transposon-encoded antibiotic resistance boosts the survival and adaptability of organisms under antibiotic pressure. This resistance can spread both within the same bacterial species and between different species, a process that is especially significant in healthcare-associated infections (HAIs), where selective outbreaks can escalate rapidly [23,24]. For example, strains of *CRKpn* not only cause nosocomial infections but also colonize patients and healthcare workers, contributing to their spread in the community [25]. Uropathogenic strains of *Kpn* can cause uncomplicated or complicated urinary tract infections (UTIs), with the latter carrying a higher risk of treatment failure and the spread of virulence/resistance determinants. Even UTIs that are considered uncomplicated (cystitis) can have adverse outcomes (liver or kidney abscesses, urosepsis, and reactivations) if inadequately treated. *CRKpn* poses a significant challenge to the management of complicated urinary tract infections (cUTIs), as these strains also exhibit significant resistance to other drugs, including aminoglycosides and fluoroquinolones [1,24]. Although in many parts of the world, several UTIs are still treated with antibiotics (e.g., fluoroquinolones) for purely clinical reasons, current treatment strategies should exclude etiological treatment without a bacteriological evaluation of the pathogen involved, even in uncomplicated forms. Although there are limited treatment options for *CRKpn*, which causes UTIs, understanding its epidemiology, molecular mechanisms of antibiotic resistance, and virulence, as well as treatment options against it, is essential.

This paper aims to provide a risk estimation tool, based on a comprehensive evaluation of patients diagnosed with *Klebsiella pneumoniae* UTIs and statistical methods, to assist clinicians in making initial treatment decisions while awaiting the phenotypic characterization of the bacterium and the final antibiotic susceptibility testing results.

## 2. Results

This study included 261 unique patients who were hospitalized and diagnosed with urinary tract infections caused by *Klebsiella pneumoniae*. The study period was two years (2022–2023). Based on the bacterial resistance identified, we divided the patients into two groups: one with carbapenem-resistant *Klebsiella pneumoniae* (*CRKpn*), consisting of 109 patients, and a control group with carbapenem-sensitive *Klebsiella pneumoniae* (*CSKpn*), consisting of 152 patients. The second group consisted of patients who were infected with *CSKpn*, including those with ESBL strains. The first group accounted for 41.76% of the total number of patients included in this study. We studied the two groups according to their demographic (sex, origin, age), clinical (length of hospital stay, type of infection, co-infections/colonizations with other pathogens, comorbidities, chronic treatments with immunosuppressive potential), anamnestic (previously documented *Kpn* infections or carriage and history of hospital admissions within the past 12 months), and laboratory data. These data are shown in Table 1.

As not all patients were admitted to the hospital with a diagnosis of UTI, and some developed symptoms during hospitalization, we evaluated the difference in the days between admission and urine culture positivity. In the *CSKpn* (control) group, the average was 2.26 days, whereas in the patients with *CRKpn*, it was 7.28 days. The difference was highly statistically significant (*p* < 0.001), which suggests that patients who were initially hospitalized for other conditions developed UTIs with *CRKpn* more frequently during their stay than those in the control group, indicating that these infections can be considered healthcare-associated infections (HAIs). Therefore, we divided the patients into two categories: those in whom *Kpn* was detected within 5 days of admission and those in whom the bacterium was identified more than 5 days after hospitalization. Following this division, we found a statistically significant difference between our groups (*p* < 0.001).

Regarding the type of infection (UTI vs. urosepsis), we did not find significant differences between the two groups, but in patients with UTIs of the upper urinary tract, we found a statistically significant difference between the two groups (*p* < 0.01). We did not find significant differences between the two groups in the location of prior infection with *Kpn* or in admission to an intensive care unit (ICU).

The simultaneous presence of other bacteria at the same site or other sites (co-infections or colonizations) did not significantly affect the studied cohorts. A total of 57 patients were found to have an infection (regardless of site) or evidence of prior *Kpn* carriage in the last 12 months, with a statistically significant difference (*p* = 0.002).

Regarding the patients’ history of admissions to other hospitals in the last 12 months, we did not observe statistically significant differences between the two groups of patients admitted to surgical wards (including urology). Major comorbidities (diabetes mellitus or neoplasia) did not affect the groups studied, nor did immunosuppressive treatments. In contrast, a recent history of invasive urinary tract procedures (including indwelling urinary catheters) in the previous 6 months strongly influenced the type of *Kpn* that was found (*p* < 0.001).

The laboratory data collected for the two groups did not show significant differences in the main laboratory constants studied (hematological, biochemical, or urinary). We found no statistically significant differences in creatinine or the estimated glomerular filtration rate (eGFR). However, when we evaluated the two groups focusing on a diagnosis of moderate to severe kidney damage or kidney failure (eGFR < 30 mL/min/1.73 m^2^) [26], we found statistically significant differences between the two groups, with a higher number of these patients being found in the *CRKpn* group (*p* = 0.017).

In the *CRKpn* group, the distribution of carbapenem-resistant genes was predominantly NDM + OXA-48-like enzymes (48.6%), followed by OXA-48-like enzymes (21.9%), KPC (19%), and NDM (10.5%).

Based on the bivariate analysis (Table 1), we identified eight statistically significant factors: male gender, the length of hospital stay, a greater number of days between admission and a positive urine culture for *Kpn*, prior infection or carriage of *Kpn*, UTI type, *Kpn* healthcare-associated infections (HAIs), recent invasive urological procedures, and stage G4 chronic kidney disease.

**Figure 1:**
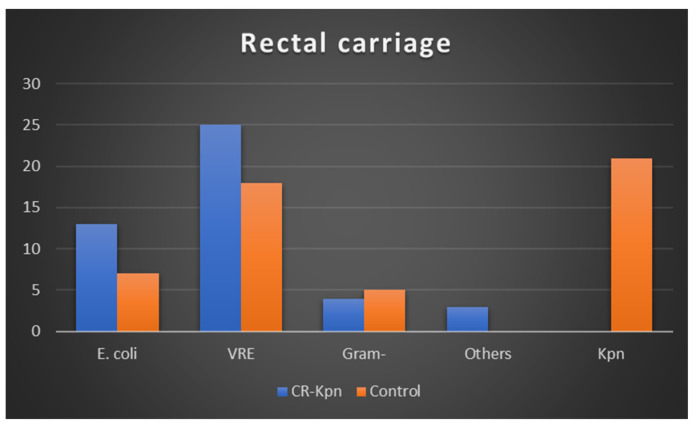
Distribution of bacteria in rectal swabs from the study groups.

We evaluated the rectal carriage in the patients studied. A total of 148 rectal specimens were collected (107 from the study group and 41 from the control group) according to the criteria described in the Materials and Methods Section. Forty-nine samples were positive in the *CRKpn* group, and forty were positive in the *CSKpn* group. We found an association between vancomycin-resistant Enterococcus (VRE) and E. coli extended-spectrum β-lactamase (ESBL) in the study group, while in the control group, we encountered *Kpn*, followed by VRE and E. coli in terms of frequency. These findings are shown in Figure 1.

We performed a bivariate analysis in both groups to evaluate the factors describing the survivors and those who died. In the table below, we summarize the data for which we found statistical significance (Table 2).

Following this analysis, we found that older age, the presence of urosepsis, and renal injury were correlated with death in both groups studied. Neutrophilia was only statistically significant in deceased patients who were infected with *CSKpn*. Higher fibrinogen levels were a “protective” factor for death, probably because of the higher incidence of hypofibrinogenemia in severe infections.

**Figure 2:**
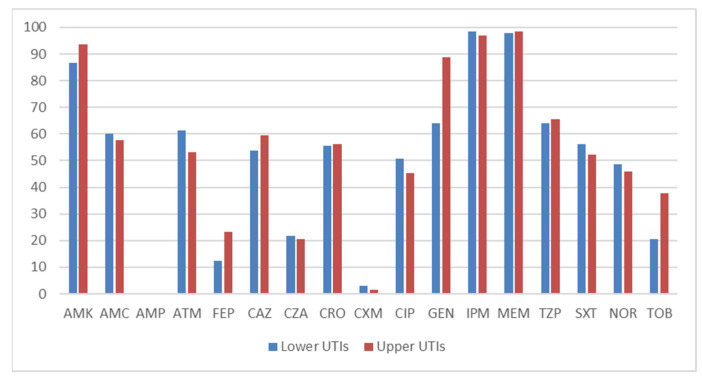
Antibiotic susceptibility of *Kpn* strains in the *CSKpn* group based on type of UTI (%).

The patients studied were divided according to their type of UTI. In the control group (*CSKpn*), 72 patients were diagnosed with lower urinary tract infections and 67 with upper urinary tract infections. In 13 cases, the type of infection could not be determined. Among the patients infected with *CRKpn*, 19 had lower urinary tract infections and 62 had upper urinary tract infections. Twenty-eight patients could not be classified into either category. We studied the antibiograms of *Kpn* within these two categories to highlight any differences. We focused solely on antibiotic susceptibility, expressed as a percentage. Thus, in the control group, we found high levels of susceptibility to carbapenems and aminoglycosides, which is consistent with data reported in recent medical publications [27]. Acceptable levels of susceptibility (above 50%) were found for ceftriaxone (CRO), ceftazidime (CAZ), trimethoprim–sulfamethoxazole (STX), and piperacillin–tazobactam (TZP). The differences between the two locations of infection (lower or higher) were small. These findings are shown in Figure 2.

**Figure 3:**
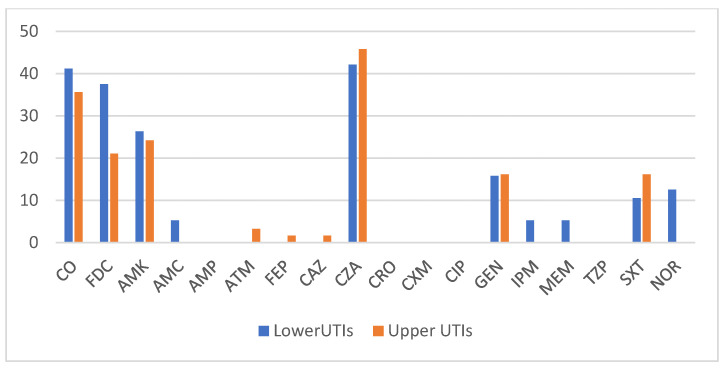
Antibiotic susceptibility levels in the CR*Kpn* group based on type of UTI (%).

For the strains found in the *CRKpn* group, as expected, the level of antibiotic susceptibility was much lower, with virtually none of the antibiotics exceeding 50%. Ceftazidim–avibactam (CZA) had the highest sensitivity, but this was still below 45% and practically only found in strains that did not contain metallo-β-lactamases, followed by colistin (CO) and cefiderocol (FDC) (Figure 3). It is worth noting that cefiderocol was not used in this cohort of patients, as it was only introduced as a therapy in Romania in 2024. Tigecycline testing was not included, because this antibiotic has no indication to treat UTIs.

In our study, we were interested in uncovering predictive factors of the presence of UTI with *CRKpn*, for which we used binomial logistic regression. Logistic regression was suitable because we wanted to estimate the probability that a UTI patient who was infected with *Kpn* belonged to one of the two studied groups in order to guide an appropriate therapeutic decision.

We included eight significant factors in the multivariate analysis (see Table 1), of which only five remained as independent factors after adjusting for the others. As can be observed in Table 3, the highest odds ratios were obtained for *Kpn* HAIs (4.193), prior *Kpn* infection or carriage (3.572), and invasive urinary procedures in the last six months (3.179). The lowest value was for male gender (2.018).

In our model, no multicollinearity was detected among the independent variables, indicating that none of them are highly correlated with one another.

Moreover, the Hosmer–Lemeshow test result was higher than 0.05, proving that the model’s estimates fit the data well, and there was no significant difference between the observed and predicted values. The Nagelkerke pseudo-R square test returned a value of 0.328, suggesting a reasonably good model.

Based on our results, we can calculate an estimation of the probability of a patient being classified into either the CS*Kpn* or CR*Kpn* group using the following equation:

$$\hat{p}=\frac{e^{\left(\beta_{0}+\beta_{1}*X_{1}+\ldots +\beta_{n}*X_{n}\right)}}{1+e^{\left(\beta_{0}+\beta_{1}*X_{1}+\ldots +\beta_{n}*X_{n}\right)}}$$

In this formula, the *βs* values are the coefficients from the logistic regression presented in column B of Table 3. We used decile analysis [28,29] to propose a procedure for quickly estimating the patients who are more likely to have a urinary infection with carbapenem-resistant *Kpn*.

**Figure 4:**
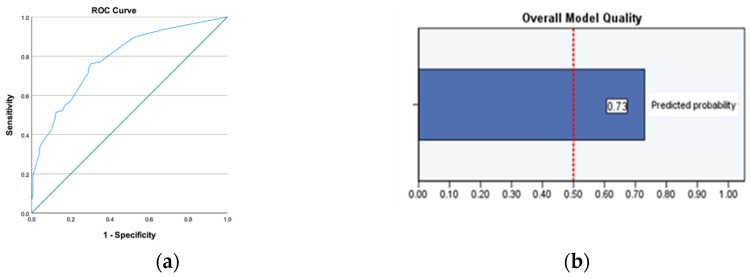
(**a**) ROC curve evaluating the performance of our prediction model for Klebsiella pneumoniae UTIs; (**b**) overall model quality and lower limit of the 95% CI.

We checked our model using ROC analysis, and the results showed a reasonable performance, with an area of 0.786, which is statistically different to the 0.5 value corresponding to a completely random choice. The ROC and model quality graphs are shown in Figure 4.

The Kolmogorov–Smirnov (K-S) metric yielded a cut-off value of 0.3424 for the probabilities within our groups (Table 4).

We also performed a decile analysis, which revealed an optimal cut-off value of around 0.463, which is in decile 4. This suggests a good discriminative ability of the model because most of the *CRKpn* cases in our dataset are above this probability value. We verified the effectiveness of the cut-off value identified based on the decile analysis by calculating the positive predictive value (PPV) and negative predictive value (NPV). For a local prevalence of 0.417, we found a PPV = 70% and an NPV = 77%.

## 3. Discussion

In this retrospective case–control study carried out in 2022–2023 on 261 patients with urinary tract infections caused by *Klebsiella pneumoniae*, we found a high incidence of *CRKpn* (41.76%), which correlates with data found in the current literature relating to Romania, where, according to the ECDC, the global percentages of *CRKpn* (including UTI) were 47.8% in 2022 and 52.8% in 2023 [30]. Among the carbapenemases, a concerning 48.6% were NDM + OXA-48-like enzymes. There is a high probability that the percentage of NDM + OXA48-like enzymes found in this study is higher than that found in general hospitals because the hospital where the study was conducted is a mono-specialty hospital that receives many cases with complicated infections from other medical or surgical (including urology) clinics. This is why we find a higher proportion of complicated infections or resistant germs than in general hospitals. Although we also deal with healthcare-associated infections (HAIs), present in all hospitals worldwide, due to our profile, we have to care for patients with HAIs who are admitted from other hospitals or who have been recently hospitalized for various conditions. However, we consider this report to be an important alert regarding the prevalence of this type of carbapenemase.

We did not find any significant differences in the number of cases between the two years studied. We also did not find any statistical significance with regard to the age of the patients, although other studies have found a higher incidence of *CRKpn* in older patients [31,32]. Evidence of previous infection or colonization with *Kpn* had a strong statistical significance (*p* = 0.02), as might be expected. The type of medical specialty to which patients were previously admitted did not show any statistical differences. In our study, the presence of *CRKpn* was not more frequent in patients with a recent history of immunosuppressive treatment.

Regarding carriage or concurrent infections with other pathogens, we found no significant differences between the two groups of patients. Regarding rectal carriage, which has been implicated by several authors as a possible source of urinary tract infection [33], we did not find the presence of *Kpn* in patients in whom we found the presence of carbapenemases. Here, we found more frequent occurrences of VRE and *E. coli*, which led us to conclude that in our group, the digestive carriage of *Kpn* did not correlate with UTIs with *CRKpn*.

Regarding the type of carbapenemases, the NDM + OXA-48-like combination was the most common, constituting almost half of the cases (48.6%). This explains the high level of antibiotic resistance of the carbapenemase strains found in our group and the major difficulties that were encountered in treating these patients during the study period.

Using logistic regression, we identified the following independent predictive factors: gender (male), time to positive specimen ≥ 5 days (HAIs), upper urinary tract infection, urological invasive procedures in the last 6 months, and prior *Kpn* infection/carriage. The model that emerged from our statistical analysis has a value of 0.786 with a 95% CI (0.730–0.842), indicating a relatively good ability to discriminate *CRKpn* cases.

However, based on the data found in the literature [23,33,34] and taking into account the statistically significant factors found in our study, we identified five factors that we considered important for the characterization of UTI patients with *CRKpn*. After a decile analysis, we found a PPV of 70% and an NPV of 77% for our model at a cut-off of 0.463. These findings indicate that probabilities above this cut-off value are better at characterizing patients in the *CRKpn* category.

**Figure A1:**
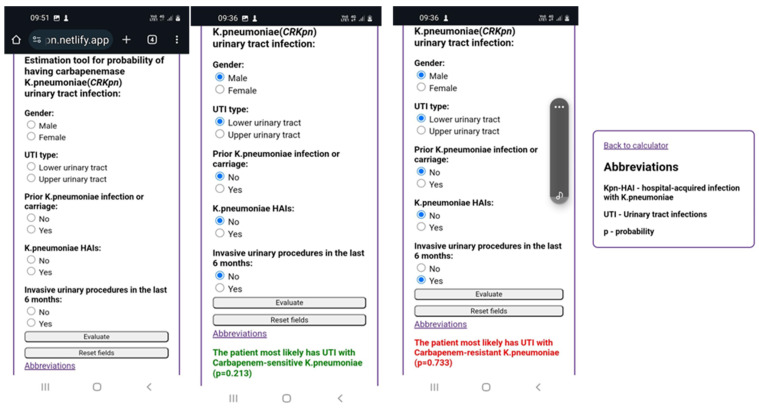
Online app for estimating the probability of having *CRKpn* according to our score.

We believe that using such a model has clinical importance, as most laboratories provide antibiogram results 24–48 h after identifying the pathogen. Additionally, many laboratories are unable to detect the presence of these carbapenemases, which could result in the patient receiving inappropriate treatment. Given the alarming increase in the presence of carbapenemases in Enterobacteriaceae, especially in *Kpn*, we argue that a tool like this could assist practitioners in prescribing antibiotics. We also designed a calculator using this model (Figure A1).

Therefore, we suggest that in hospitals with a high prevalence of metallo-β-lactamases (NDM, NDM + OXA-48-like enzymes), a new therapeutic option (ceftazidime–avibactam plus aztreonam or cefiderocol in monotherapy) should be considered if the estimated probability is higher than the cut-off value of 0.463, thereby avoiding the use of carbapenems [35,36,37].

If there is a higher local prevalence of other types of carbapenemases, other therapeutic options may be used. For example, ceftazidime–avibactam, meropenem–vaborbactam, and imipenem–relebactam can be used as monotherapies or, preferably, in combination with other antibiotics [38]. If the probability is less than 0.463, the use of carbapenems or various combinations of them (e.g., with aminoglycosides) can be considered. Since it is difficult to calculate probabilities in real-life medical settings, we suggest using the tool described in Appendix A, which is an online calculator.

### Strengths and Limitations

This paper proposes using a clinical score for the evaluation of urinary tract infections with CRKpn, which can be useful for clinicians in the period between the identification of the germ and the final result of the antibiogram. For *CRKpn* infections, regardless of their location, several scores have been presented in the literature relating to laboratory testing or clinical outcomes. However, only a few studies propose an estimation tool based on machine learning to guide therapeutic decision making, whereas we developed a model using a classic statistical approach for UTIs only [39,40,41]. We drew on the decile analysis procedure to obtain an improved estimated probability point cut-off for our dataset.

We are aware that the present study has several limitations, such as the small size of the group studied, the lack of a validation group, and some of the peculiarities that we encountered: the higher prevalence of CRKpn compared with other studies and the high number of metallo-β-lactamase infections. Other aspects could interfere with our model, e.g., recall bias (a chance of inaccurate reporting when patients are asked to remember past exposures). Also, the cut-off chosen for this model depended on the peculiarities of the group studied, which may not be found in other similar studies.

## 4. Materials and Methods

### 4.1. Patient Data Collection

We conducted this retrospective case–control study on a group of 261 patients who were admitted to the “Dr Victor Babes” Hospital for Infectious and Tropical Diseases, Bucharest, Romania. This study was conducted between 1 January 2022 and 31 December 2023.

All patients who were diagnosed with a urinary tract infection with *Klebsiella pneumoniae* during this period were included, regardless of whether the diagnosis was made at admission or during hospitalization. Patients were diagnosed on the basis of clinical, paraclinical, and laboratory data. The inclusion criteria were adult patients (over 18 years of age) diagnosed with a urinary tract infection with *Kpn* during the study period. The exclusion criteria were age under 18 years, patients with asymptomatic bacteriuria detected by means of *Kpn*, and without evidence of infection.

Depending on the presence of carbapenemases, we divided the two groups into carbapenem-resistant *Kpn* (*CRKpn*) and carbapenem-sensitive *Kpn* (*CSKpn*), with the second group being the control group. We assessed the antibiotic susceptibility in each of the two groups. For both groups, we extracted demographic data (sex, age, place of origin) from the medical records. We studied the available medical history: comorbidities and previous hospitalizations in the last year, which we divided into urology departments, surgical departments (regardless of type), and non-surgical departments.

We analyzed patients who had undergone previous invasive maneuvers in the previous six months. Regarding those with invasive urological maneuvers in the recent history, these were performed in other hospitals, with a significant part of the patients being referred to our hospital for persistent UTIs or in whom CRKpn infections had already been diagnosed.

Patients were evaluated according to clinical aspects: severity, type of UTI (upper urinary tract vs. lower urinary tract), concomitant co-infections or carriers of MDR germs (with a focus on rectal carriers), etiological treatment, and patient outcome. In the case of “upper UTIs”, we considered patients who had a diagnosis of acute pyelonephritis or those with infected ureteral catheters. We also included in this group patients who had a record of unspecified urinary tract infection but who had clinical signs suggestive of upper UTIs (high fever, chills, back pain, or pyuria). In the patients included in the “lower UTIs” category, we considered the diagnosis recorded by the attending physician or the characteristic symptomatology. There were patients who were considered “lower UTIs” upon admission but evolved to an upper urinary tract infection and therefore were categorized in the second category. In the case of patients with urosepsis, based on the history prior to admission, we classified them into the two categories based on clinical criteria. According to the hospital’s internal protocol, we collect rectal swabs from all patients who have been hospitalized in the last 30 days in other clinics and have undergone invasive procedures or received antibiotic treatments, or from those who are transferred to the ICU from other departments of the hospital.

We assessed the kidney function status in the patients, based on both their medical history and the analyses that were performed during their present hospitalization. For this, we distinguished an estimated glomerular filtration rate (eGFR) value below 30 mL/min/1.73 m^2^, which corresponds to moderate to severe kidney damage or kidney failure. We considered in our study the mean creatinine value for each patient per hospitalization episode, and that is why we did not set out to make a distinction between acute and chronic renal injury.

In the two groups, we evaluated the possibility of urinary tract infection with *Kpn* associated with medical activity (nosocomial). For this, we separated the patients who had a positive urine culture for *Kpn* five or more days after admission [42]. We chose this “cut-off” for patients who were admitted with a different pathology and did not have a urinary infection at admission. These patients were considered as having HAIs.

### 4.2. Microbiological Evaluation

Bacterial species identification was performed using several automated identification systems (using the exoenzymatic properties of bacteria/VITEK2C), mass spectrometry (MALDI-TOF/Matrix-Assisted Laser Desorption/Ionization–Time of Flight Mass Spectrometry/BRUKER Daltonics GmbH, Karlsruhe, Germany). The bacterial strains were screened for antibiotic resistance phenotypes using chromogenic media, with results being confirmed using standard methods. Antibiotic susceptibility testing (antibiogram) was performed using phenotypic techniques. The routine antibiotic susceptibility testing included disk diffusion (standardized disk diffusion method/Kirby Bauer), quantitative methods (MIC) in automated VITEK2C systems, the concentration gradient method (E-Test), and the microdilution method in Mueller–Hinton broth (International Standard Reference for non-fastidious aerobic bacteria/EUCAST).

In 2022, we performed quantitative antibiograms (CMI) in an automatic VITEK2C system. In 2023, we performed quantitative antibiograms using the Bullion Microdilution Method in the automatic Micronaut/Bruker system. We only used the concentration gradient method, using E-Test strips (BioMerieux) for the synergism test between ceftazidime–avibactam and aztreonam. We did not routinely test the same strain using both quantitative methods (VITEK2C and MCRONAUT) and did not encounter any discrepancies. The final result of the test was the one obtained by means of microdilutions in broth (Micronaut/Bruker).

The testing of bacterial isolates and the interpretation of the test results were performed according to the EUCAST standard recommendations, including cefiderocol [43]. Resistance mechanisms were detected by means of phenotypic methods using screening tests for carbapenemase production: rapid immunochromatographic tests and double-disk synergy tests (DDSTs). Confirmation was performed using genotypic techniques for antibiotic resistance testing (GeneXpert Carba-R), which is a qualitative in vitro diagnostic method for the rapid and differentiated detection of gene sequences that are associated with resistance to carbapenems in Gram-negative bacteria, such as blaKPC, blaNDM, blaVIM, blaOXA-48, and blaIMP-1 [44]. Using the rectal carriage procedure, we detected the presence of certain multidrug-resistant organisms in the gastrointestinal tract. This procedure is often carried out to identify carriers who may not have active infections but could spread the bacteria within healthcare settings.

### 4.3. Statistical Analysis

Demographics, medical history, clinical and paraclinical aspects, length of hospital stay, and patient outcomes were collected from the hospital informatics system and stored in an Office Excel dataset for further processing. The Shapiro–Wilk and Kolmogorov–Smirnov tests were used to assess the normality of continuous data. In the bivariate analysis, the ANOVA test was used for continuous data to determine significant differences between the two categories. The results for continuous variables are presented as the average and 95% CI, and for categorial variables, they are presented as the number and percentage of cases in each category. Associations between two categorical variables were tested using Pearson’s chi-squared test and Yates’ correction for a 2 × 2 table or Fisher’s exact test for a table with more than 2 × 2 categories. Relevant factors identified in the bivariate analysis were selected for multivariate analysis (binomial logistic regression) to determine independently associated factors. The model obtained from binomial logistic regression was further tested using the ROC, and the cut-off value was obtained using the Kolmogorov–Smirnov test (K-S statistics). To optimize the cut-off value in our dataset, we used the decile analysis method. Decile analysis simplifies the process of selecting an optimal cut-off for data classification by dividing the data into ten equal groups (deciles). The cases are sorted in ascending or descending order, allowing the analyst to identify a suitable cut-off point. In our case, we performed logistic regression to identify significant variables, calculated probabilities for each case, and applied decile analysis by sorting these probabilities in descending order. This approach helped us determine a cut-off to guide strategies for patients with *CRKpn* [28,29].

The positive and negative predictive values of the score for the optimal values were determined to verify the cut-off values. Microsoft Excel (version 2021) was used for data collection and graphing, and IBM SPSS Statistics v26 was used for data processing.

## 5. Conclusions

The aim of the present work was to evaluate the characteristics of patients who were hospitalized in a tertiary hospital in Romania over a period of 2 years with UTIs with *CRKpn*. Based on the statistical analyses and identification of predictive factors, we proposed a method for assessing the presence of *CRKpn* in patients in whom *Klebsiella pneumoniae* was identified as the etiology of their UTI.

The decile analysis indicates a reasonable predictive capacity of the model, with a high concentration of positive cases in the upper deciles, suggesting that the model effectively differentiates between cases with high and low probabilities.

The fact that the upper deciles (1–4) have a higher proportion of positive cases validates the model’s ability to distinguish between different risk levels, thereby strengthening the practical applicability of the cut-off value.

Using this model, clinicians could consider treatment options based on probabilistic data, pending the final results of the antibiogram. Although we are aware of the limitations of this study, we believe that the model could be improved and validated by further studies in the future.

## Figures and Tables

**Table 1:** *CRKpn* vs. *CSKpn* group comparison.

Variable	*CSKpn* *n* = 152	*CRKpn* *n* = 109	*p*-Value
Study Year (*n*, %)			0.115
2022	55 (36.2)	50 (45.9)	
2023	97 (63.8)	59 (54.1)	
Gender (n, %)			0.002
Male	49 (46.7)	56 (53.3)	
Female	103 (66.0)	53 (34.0)	
Rural or Urban residence (n, %)			0.181
Rural	20 (13.2)	21 (19.3)	
Urban	132 (86.8)	88 (80.6)	
Age (average, 95% CI)	68.3 (65.41–71.34)	70.5 (67.92–73.14)	0.304
Days in hospital (average, CI95%)	14.03 (12.20–15.85)	25.31 (21.51–29.10)	<0.001
Days between admission and *Kpn* confirmation (average, CI95%)	2.26 (1.14–3.37)	7.28 (4.57–9.73)	<0.001
*Kpn* HAIs * (yes, %)	17 (11.2)	31 (28.4)	<0.001
Type of infection			0.101
UTI	122 (80.3)	78 (71.6)	
Urosepsis	30 (19.6)	31 (28.4)	
UTI type			<0.001
Lower UTI	72 (51.8)	19 (23.5)	
Upper UTI	67 (48.2)	62 (76.5)	
Diagnosis—UTI			0.340
Main diagnosis	68 (44.7)	54 (50%)	
Secondary diagnosis	70 (46.1)	50 (46.3)	
Location of prior *Kpn* infection			0.103
Urinary	18 (100.0)	19 (86.4)	
Other	0 (0.0)	3 (13.6)	
ICU admission (yes, %)	10 (41.7)	14 (58.3)	0.084
Days in ICU (average, CI95%)	9.4 (3.19–15.61)	11.1 (7.00–15.31)	0.594
Carbapenemase type (n, %)	-		
Oxa48	-	23 (21.9)	-
NDM	-	11 (10.5)	-
KPC	-	20 (19.0)	-
NDM + OXA-48-like enzyme	-	51 (48.6)	-
Bacterial co-infections/colonizations	43 (51.2)	41 (48.8)	0.112
Bacterial type (yes, %)			0.250
G-negative	27 (65.9)	27 (65.9)	
G-positive	6 (14.6)	10 (24.4)	
G-negative and G-positive	5 (12.2)	4 (9.8)	
Prior *Kpn* infection/carriage (yes, %)	16 (18.0)	41 (36.6)	0.002
Passing through a urology ward (yes, %)	25 (16.4)	17 (15.6)	0.854
History of surgery (yes, %)	29 (19.1)	20 (18.3)	0.882
Passing through other medical wards (yes, %)	92 (60.5)	54 (49.5)	0.078
Comorbidities			
Neoplasm (yes, %)	40 (26.3)	31 (28.4)	0.704
Diabetes mellitus (yes, %)	46 (30.3)	32 (29.4)	0.875
Treatments and interventions			
Corticotherapy (yes, %)	3 (10.0)	5 (4.5)	0.373
Immunosuppression (yes, %)	10 (6.6)	10 (9.2)	0.437
Chemotherapy (yes, %)	17 (11.2)	10 (9.3)	0.616
Monoclonal antibodies (yes, %)	4 (2.6)	2 (1.9)	0.680
Radiotherapy (yes, %)	6 (3.9)	1 (0.9)	0.135
Invasive urinary procedures < 6 mo. (yes, %)	61 (40.1)	78 (71.6)	<0.001
Urinary catheters (yes, %)	26 (17.1)	26 (24.1)	0.166
Laboratory results			
Nitrituria (yes, %)	53 (41.1)	36 (39.6)	0.820
Leukocytes (average, CI95%)	11,516 (10,245.9–12,786.2)	11,827 (10,316.6–13,338.91)	0.755
Neutrophils (average, CI95%)	9273 (7894.2–10,653.1)	9349 (7882.2–10,816.8)	0.942
C-reactive protein (CRP) (average, CI95%)	11.3 (5.08–17.65)	8.33 (6.83–9.83)	0.426
Fibrinogen (average, CI95%)	529 (495.8–563.4)	541 (501.2–581.0)	0.664
Creatinine (average, CI95%)	1.32 (1.14–1.50)	1.61 (1.34–1.88)	0.070
eGFR (average, CI95%)	69.8 (63.86–75.80)	61.6 (55.14–68.17)	0.072
eGFR-30 ** (< 30, %)	18 (11.8)	25 (22.9)	0.017

* Kpn HAIs: *Kpn* cultures were positive more than 5 days after admission. ** eGFR-30: estimated glomerular filtration rate was <30 mL/min/1.73 m^2^ compared with ≥30 mL/min/1.73 m^2^.

**Table 2:** Summary of bivariate analysis in the studied groups for survivals vs. deaths.

Variable	*CRKpn*			*CSKpn*		
	Survivals	Deaths	*p*-Value	Survivals (n = 140)	Deaths	*p*-Value
	n = 79	n = 30			(n = 12)	
Age at admission (average)	68.7	75.2	0.026	67	83.8	0.002
	(65.49–71.98)	(71.39–79.14)		(63.93–70.14)	(77.8–89.8)	
Type of infection (n, %)			<0.001			<0.001
UTI	65 (82.3)	13 (43.3)		118 (84.3)	4 (33.3)	
Urosepsis	14 (17.7)	17 (56.7)		22 (15.7)	8 (66.7)	
UTIs (n, %)			0.494			0.003
Lower UTI n = 91	15/59 (25.4)	4/22 (18.2)		71/128 (55.5)	1/11 (9.1)	
Upper UTI n = 129	44/59 (74.6)	18/22 (81.8)		57/128 (44.5)	10/11 (90.9)	
Invasive urinary procedures < 6 mo. (n, %)	53 (67.1)	25 (83.3)	0.093	53 (37.9)	8 (66.7)	0.051
Leukocytes (blood) (average, 95% CI)	12,491	10,080	0.159	10,914	7280	0.001
	(10,539.12–14,443.91)	(8132.31–12,027.68)		(9697–12,131)	(11,175–25,891)	
Neutrofils (blood) (average, 95% CI)	9875	7963	0.25	8670	16,316	0.003
	(7969.11–11,782.78)	(6105.19–9821.47)		(7305–10,034)	(9490–23,142)	
Fibrinogen (average, 95% CI)	565	467	0.034	540	406	0.029
	(6.20–9.93)	(6.50–11.53)		(505.0–576.6)	(313.41–499.49)	
Creatinin (average, 95% CI)	1.44	2.07	0.038	1.22	2.52	<0.001
	(1.17–1.70)	(1.36–2.78)		(1.06–1.38)	(1.16–3.88)	

**Table 3:** Summary of multivariate logistic regression.

Predicting Factors *	B (β)	*p*-Value	Odds Ratio	95% CI for Odds Ratio	
				Lower	Upper
Invasive urinary procedures <6 mo.	1.157	<0.001	3.179	1.761	5.739
*Kpn* HAIs (yes)	1.433	<0.001	4.193	2.076	8.469
Prior Kpn infection/carriage	1.273	0.001	3.572	1.683	7.583
Gender *	0.702	0.019	2.018	1.125	3.620
UTI type *	1.101	0.009	3.008	1.317	6.867
Constant	−2.007	<0.001	0.134	-	-

Note: * Gender is measured as male compared with female; UTI type is measured as upper UTI compared with lower UTI; and all other factors are measured as “yes” compared with “no”.

**Table 4:** K-S statistics establishing cut-off values.

Classifier Evaluation Metrics		
Test Result Variable(s): Predicted Probability		
Gini Index	K-S Statistics	
	Max K-S *	Cut-Off
0.572	0.459	0.3424

* Maximum Kolmogorov–Smirnov (K-S) metric.

## Data Availability

The corresponding author can provide the data used in this study upon reasonable request.

## Appendix A

**Table A1:** Steps for obtaining our model for discriminating between patients with *CSKpn* and *CRKpn*.

Step 1	We collected a dataset containing variables corresponding to the purpose of this study. For a binary analysis, we ensured that the control–case column only had binary values.
Step 2	We performed a logistic regression analysis that helped to distinguish between patient categories of interest. We used logistic regression because of the simplicity of the interpretation of the logistic regression coefficients. Using other classification algorithms may not result in easy-to-interpret coefficient/weight values. Any current statistics software also provides a classification table for a cut-off probability of 0.5 along with its logistic regression coefficients. Using the logistic regression coefficients, we calculated the expected probabilities for each patient in our sample. Performing ROC analysis, we obtained a cut-off value.
Step 3	We drew on the decile analysis procedure to obtain an improved estimated probability cut-off for our dataset. To accomplish this, we sorted the estimated calculated probabilities, along with the already known observed case–control values, in descending order. We then divided our dataset into ten bins, with each bin accounting for 10% of the entire dataset.
Step 4	By inspecting each bin and looking at the number of observations in each, a medical team of specialists decided the cut-off for the estimated probability value. The cut-off was a trade-off value defined by the specialist and specific to the analyzed dataset. In our dataset, this cut-off value was chosen as a compromise between the PPV (positive predictive value) and NPV (negative predictive value) in an effort to accurately estimate as many patients as possible in the CRKpn group.
Step 5	Based on the defined cut-off value, we could compute the sensitivity, specificity, positive predictive value, and negative predictive value of our dataset.
Step 6	We developed a one-page website that utilizes the significant independent variables identified through logistic regression. The site will calculate an estimated probability based on the inputs provided by physicians. On the interface, specialists will be able to view the calculated probability of a patient belonging to the CRKpn group.

**Table A2:** Summary of decile analysis.

	Decile	Decile	Decile	Decile	Decile	Decile	Decile	Decile	Decile	Decile
	1	2	3	4	5	6	7	8	9	10
Cases/decile	26	26	26	26	26	26	26	26	26	27
*CRKpn*	25	18	14	15	11	14	3	2	1	6
*CSKpn*	1	8	12	11	15	12	23	24	25	21
*CRKpn*/decile (%)	96.15	69.23	53.84	57.69	42.30	53.84	11.53	7.69	3.84	22.22
Cumulative CRKpn (%)	22.93	39.44	52.29	66.05	76.14	88.99	91.74	93.57	94.49	100
Probability range	0.783–0.974	0.641–0.783	0.562–0.641	0.463*–0.562	0.324**–0.463	0.299–0.324	0.213–0.299	0.118–0.213	0.118	0.118

* Corresponds to probability cut-off value of 0.463, obtained from decile analysis. ** Corresponds to probability cut-off value of 0.324, obtained from ROC analysis. Explanations: *CRKpn*/decile (%): the percentage of CRKpn encountered in each decile. Cumulative CRKpn (%): cumulative percentages of CRKpn increasing from one decile to another. Example: 66% of positive cases are found between deciles 1–4. Probability range: the probability limits encountered in each decile.
